# Exome sequencing of Saudi Arabian patients with ADPKD

**DOI:** 10.1080/0886022X.2019.1655453

**Published:** 2019-09-05

**Authors:** Fahad A. Al-Muhanna, Abdullah M. Al-Rubaish, Chittibabu Vatte, Shamim Shaikh Mohiuddin, Cyril Cyrus, Arafat Ahmad, Mohammed Shakil Akhtar, Mohammad Ahmad Albezra, Rudaynah A. Alali, Afnan F. Almuhanna, Kai Huang, Lusheng Wang, Feras Al-Kuwaiti, Tamer S. Ahmed Elsalamouni, Abdullah Al Hwiesh, Xiaoyan Huang, Brendan Keating, Jiankang Li, Matthew B. Lanktree, Amein K. Al-Ali

**Affiliations:** a Department of Internal Medicine, King Fahd Hospital of the University, Al-Khobar, Imam Abdulrahman Bin Faisal University, Dammam, Saudi Arabia;; b Department of Clinical Biochemistry, College of Medicine, Imam Abdulrahman Bin Faisal University, Dammam, Saudi Arabia;; c Department of Nephrology, King Fahd Military Medical Complex, Dhahran, Saudi Arabia;; d Department of Radiology, King Fahd Hospital of the University, Al-Khobar, Imam Abdulrahman Bin Faisal University, Dammam, Saudi Arabia;; e BGI-Shenzhen, Shenzhen, China;; f BGI-Shenzhen, China National GeneBank, Shenzhen, China;; g Department of Computer Science, City University of Hong Kong, Hong Kong, Hong Kong;; h Cardiovascular Institute, University of Pennsylvania School of Medicine, Philadelphia, PA, USA;; i Division of Nephrology, McMaster University, Hamilton, Canada

**Keywords:** ADPKD, PKD1, Saudi Arabia, CFTR, EGF, TSC2

## Abstract

**Purpose:** Autosomal dominant polycystic kidney disease (ADPKD) is characterized by progressive development of kidney cysts and enlargement and dysfunction of the kidneys. The Consortium of Radiologic Imaging Studies of the Polycystic Kidney Disease (CRISP) cohort revealed that 89.1% had either a *PKD1* or *PKD2* mutation. Of the CRISP patients with a genetic cause detected, mutations in *PKD1* accounted for 85%, while mutations in the *PKD2* accounted for the remaining 15%. Here, we report exome sequencing of 16 Saudi patients diagnosed with ADPKD and 16 ethnically matched controls.

**Methods:** Exome sequencing was performed using combinatorial probe-anchor synthesis and improved DNA Nanoballs technology on BGISEQ-500 sequencers (BGI, China) using the BGI Exome V4 (59 Mb) Kit. Identified variants were validated with Sanger sequencing.

**Results:** With the exception of GC-rich exon 1, we obtained excellent coverage of *PKD1* (mean read depth = 88) including both duplicated and non-duplicated regions. Of nine patients with typical ADPKD presentations (bilateral symmetrical kidney involvement, positive family history, concordant imaging, and kidney function), four had protein truncating *PKD1* mutations, one had a *PKD1* missense mutation, and one had a *PKD2* mutation. These variants have not been previously observed in the Saudi population. In seven clinically diagnosed ADPKD cases but with atypical features, no *PKD1* or *PKD2* mutations were identified, but rare predicted pathogenic heterozygous variants were found in cystogenic candidate genes including *PKHD1, PKD1L3, EGF, CFTR*, and *TSC2*.

**Conclusions:** Mutations in PKD1 and PKD2 are the most common cause of ADPKD in Saudi patients with typical ADPKD.

**Abbreviations:** ADPKD: Autosomal dominant polycystic kidney disease; *CFTR*: Cystic fibrosis transmembrane conductance regulator; *EGF*: Epidermal growth factor; MCIC: Mayo Clinic Imaging Classification; PKD: Polycystic kidney disease; *TSC2*: Tuberous sclerosis complex 2

## Introduction

Polycystic kidney disease (PKD) is the most common inherited multi-systemic disease characterized by the progressive development of kidney cysts with consequent enlargement of the kidney and progression toward end-stage renal disease (ESRD) [1,2]. Kidney cysts range in size from microscopic to many centimeters in diameter, leading to inflammation, regional ischemia, cytokine release, tubular obstruction, and subsequent loss of kidney function. Two forms of PKD include autosomal dominant polycystic kidney disease (ADPKD) and autosomal recessive polycystic kidney disease (ARPKD) that are typically distinguishable by their pattern of inheritance, as well as age of onset, hepatic fibrosis, arterial hypertension, kidney morphology, and cyst location [3]. ADPKD is the most prevalent inherited form of kidney disease with an incidence of 1:500 to 1:1000 individuals and is observed in approximately 7%–10% of patients with ESRD [4,5]. Extra-renal formation of cysts, mainly in the liver and pancreas, abnormalities in connective tissues, and aortic and intracranial aneurysms have been reported in ADPKD patients [6]. Hypertension is prevalent among ADPKD patients and usually precedes loss of glomerular filtration rate [7]. Additional cystic kidney phenotypes exist that could be misdiagnosed as ADPKD including multiple simple cysts, autosomal dominant tubulointerstitial nephritis, tuberous sclerosis complex, medullary sponge kidney, *HNF1B* nephropathy, nephronopthisis, and others [8]. Genetic testing could be useful to clarify the diagnosis in patients with features inconsistent with typical ADPKD.

Mutations in two genes, *PKD1* and *PKD2*, are implicated in ADPKD, but despite comprehensive screening, up to 15% of suspected ADPKD patients have no mutation detected. Mutations in two additional genes, *GANAB* and *DNAJB11,* may account for an additional 1% of ADPKD patients [9,10]. *PKD1* codes for a large integral glycoprotein, polycystin 1, with an extracellular and intracellular domain suggesting that it plays a role in cell-to-cell interaction and in cellular signaling [11]. *PKD2* encodes a smaller transmembrane protein, polycystin 2, which appears to be a divalent cation channel; however, this theory has been put into question by a recent study [12]. In the Consortium of Radiologic Imaging Studies of Polycystic Kidney Disease (CRISP) cohort, 89.1% had either a *PKD1* or *PKD2* mutation. Of CRISP patients with a genetic cause detected, mutations in *PKD1* accounted for 85%, while mutations in the *PKD2* accounted for the remaining 15% [11]. In clinically ascertained cohorts, the proportion of *PKD2* mutations appears greater, representing 30% of patients [13]. Over 2,300 pathogenic *PKD1* and 300 pathogenic *PKD2* germline mutations have been identified, though primarily in European populations [12]. Although mutations in *PKD1* and *PKD2* appear fully penetrant, variable expressivity exists with disease severity varying between family members with the same mutation, implying the possibility of additional modifier genes [14].

In the last ten years, exome sequencing of 180,000 exons has been proven to be a powerful technique for the identification of rare genetic variants. The *PKD1* gene contains 46 exons with a large duplicated region spanning exons 1 to 33 [12]. The duplicated regions share a high degree of sequence identity with six pseudogenes adjacent to *PKD1* on chromosome 16. *PKD2* is a relatively smaller gene with 15 exons [12]. Mutation screening of *PKD1* with long-range polymerase chain reaction (PCR) to avoid the pseudogene sequence is technically challenging, laborious, and costly [15]. Recent efforts have successfully employed high-throughput targeted gene panel sequencing-based mutation screening of *PKD1*, while others have reported poor read depth and coverage of *PKD1* with exome-based approaches [16,17]. We attempted exome sequencing in 16 clinically diagnosed ADPKD patients and 16 normal controls from the Eastern Province of Saudi Arabia to identify mutations leading to the development of ADPKD in our population.

## Methods

### Patient selection

**Figure 1.**
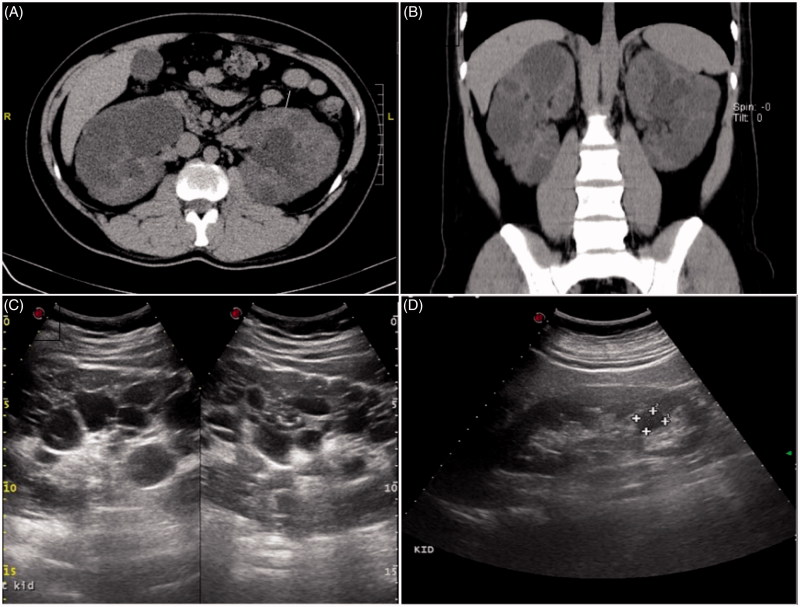
Representative computed tomography image of ADPKD patients. A and B are computed tomography imaging of (A) 38-year-old male patient P1 and (B) 49-year-old male patient P3. Kidney ultrasound images of P1 (C) and P3 (D).

This case-control study included 16 ADPKD probands ascertained in the nephrology clinic at King Fahd Hospital of the University, Al-Khobar and King Fahd Military Medical Complex, Dhahran, in the Eastern Province of Saudi Arabia. Patient demographic and clinical characteristics are provided in Table 1. We attempted to exclude all patients with syndromic causes of multiple renal cysts, such as tuberous sclerosis, von Hippel-Lindau disease, and familial polythelia with multiple renal cysts from the study. Clinical diagnosis of ADPKD was obtained using computerized tomography (CT) imaging (Figure 1). Estimated glomerular function (eGFR) was calculated from serum creatinine using the CKD-EPI equation. ADPKD diagnosis was based upon the unified Pei-Ravine diagnostic criteria when family history was present [18]. The presence of multiple cysts with dilated collecting ducts, enlarged renal outlines, increased renal echogenicity, loss of corticomedullar differentiation, and positive family history was documented. In three patients, it was not possible to confirm whether there was a positive family history of ADPKD due to unavailability of parental medical records. Total kidney volume was calculated using the ellipsoid equation, and Mayo Clinic imaging classification (MCIC) was calculated using their web-based tool [19]. Patients were evaluated for typical features of ADPKD including the following: bilateral symmetrical involvement of both kidneys (i.e., MCIC class 1), the presence of positive family history, and concordance between kidney imaging and rate of loss of kidney function. A total of 16 healthy controls with no family history of ADPKD or related conditions were randomly selected from the Eastern Province population. This study was approved by the Ethical Committee of Imam Abdulrahman Bin Faisal University in accordance with the 1964 Helsinki Declaration and its later amendments. Informed written consent in English, with a verified translation in Arabic, was obtained from all participants in accordance with the Institutional Review Board (IRB # 2014–01‐274).

**Table 1.** Clinical Characteristics and sequencing results in Saudi ADPKD patients.

Patient ID	Age	Sex	Height (m)	Hypertension	Age at ADPKD diagnosis	CKD stage	eGFR (mL/min/1.73m^2^)	# of affected family members	Total kidney volume	MCIC	Typical* presentation	Gene	cDNA	Protein
P1	38	M	1.61	Yes	26	4	26	5	604	1B	Yes	*PKD1*	c.9616C>T	p.Gln3206*
P2	44	F	1.57	No	NA	5	6	7	530	1B	No	*---*		
P3	49	F	1.65	Yes	31	5	15	2	2090	1C	Yes	*PKD1*	c.10540 C>T	p.Q3514*
P4	33	F	1.65	Yes	28	3	55	2	1236	1D	Yes	*---*		
P5	49	M	1.59	No	16	5	<15	2	2198	1D	Yes	*PKD2*	c.1390C>T	p.Arg464*
P6	60	F	1.53	No	50	5	<15	1	1532	1C	Yes	*PKD1*	c.6487C>T	p.Arg2163*
P7	39	M	1.77	No	32	3	40	2	1995	1D	Yes	*PKD1*	c.6487C>T	p.Arg2163*
P8	74	M	1.59	Yes	68	1	97	0	927	1B	No	*---*		
P9	54	M	1.68	No	NA	5	11	1	384	1A	No	*---*		
P10	37	M	1.80	Yes	35	1	94	1	119	1A	Yes	*PKD1*	c.12436G>A	p.Val4146Ile
P11	27	M	1.60	No	26	5	12	3	251	1A	No	*EGF*	c.1097G>A	p.Gly366Asp
P12	49	F	1.60	Yes	49	5	<15	1	270	1A	No	*PKD1L3*	c.3503C>G	p.Ser1168*
P13	36	M	1.74	Yes	NA	3	44	2	2036	1D	Yes	*CFTR*	c.358G>A	p.Ala120Thr
P14	48	M	1.65	Yes	38	3	43	5	3120	1D	Yes	*PKHD1*	c.5725C>T	p.Arg1909Trp
P15	52	M	1.65	Yes	47	5	11	0	297	1A	No	*CFTR*	c.358G>A	p.Ala120Thr
P16	46	M	1.69	No	45	3	40	0	300	1A	No	*TSC2*	c.2954C>T	p.Ala985Val

ADPKD Mayo Clinic Imaging Classification, MCIC (www.mayo.edu/research/documents/pkd-center-adpkd-classification/).

*Typical presentation includes bilateral symmetrical kidney involvement, positive family history, and concordant imaging results and rate of eGFR decline.

### Exome sequencing and data analysis

Peripheral blood samples were collected in EDTA tubes and stored at 4 °C before extraction of genomic DNA using Gentra Puregene Blood kits (Qiagen, USA) according to the manufacturer's protocol. BGISEQ-500 Paired Library preparation was performed in accordance with the instruction protocol (BGI, China) described elsewhere [20]. We performed whole exome sequencing of all samples on the combinatorial probe-anchor synthesis and improved DNA Nanoballs technology-based BGISEQ-500 sequencers (BGI, China) using the BGI Exome V4 (59 Mb) Kit (BGI, China). Exome data were mapped to the human reference genome (NCBI build 37.1, UCSC hg19) using Burrows-Wheeler Aligner (BWA-MEM, version 0.7.10) [21]. Variant calling was performed using Genome Analysis Tool Kit (GATK, version 3.3) after removal of the duplicate reads by Picard [22]. All variants were annotated by ANNOVAR and Variant Effect Predictor (VEP) [23]. Variants with a read depth > 20 were extracted for further analysis. The variants which were identified through the above pipeline were further filtered to eliminate benign variants with MAF (minor allele frequency)>0.1% in the 1,000 Genomes Project, dbSNP, exome aggregation consortium (ExAC), ESP-6500, and internal BGI databases containing exome data on >100 000 subjects [24–26]. Finally, the candidate variants were checked against the Human Gene Mutation Database (HGMD), the Mayo PKD Mutation Database, and Clinvar, if mutations had been previously published [27–29].

### Validation by Sanger sequencing

Sanger sequencing was used to validate all mutations identified through high-throughput exome sequencing (Supplementary Figure 1). PCR primers were designed with Primer3. The first step in the PCR was performed at 95 °C for 5 min, then followed by 30 cycles of denaturation at 95 °C for 30 s, annealing at 55 °C for 30 s, extension at 72 °C for 30 s, and a final extension at 72 °C for 7 min. The PCR products were sequenced using ABI 3730 xl DNA Analyzer, and the results were compared with the reference sequence (UCSC hg19).

## Results

The majority of recruited patients were male (69%), with a mean age of 46 years and a mean age of diagnosis of 34 years. The mean age of the female patients was 50 years, with a mean age of diagnosis of 36 years. Hypertension was reported in nine of the total patient number. Nine of the 16 patients had typical ADPKD with MCIC class 1 imaging findings, positive family history, and consistent MCIC and kidney function. Seven patients were found to have atypical ADPKD features including the following: Six of these seven patients had evidence of renal atrophy with reduced eGFR (P2, P9, P11, P12, P15, and P16), and three of the patients had no known family history (P8, P15, and P16). After reviewing the radiological liver images of patients, no cysts were found to be present in the liver.

Exome sequencing was performed in 16 patients clinically diagnosed with ADPKD and 16 ethnically matched controls. Of the 59 million bases captured, approximately 50%, 62.8%, 77.3%, 90.5%, and 96.7% of bases were sequenced at a depth of at least 200×, 150×, 100×, 50×, and 20×, respectively. The mean read depth for *PKD1* was 88 (standard deviation = 49), with adequate coverage of all exons of the duplicated and non-duplicated region with the exception of GC-rich exon 1 (Supplementary Table 1).

### Mutations identified in patients with ADPKD

In the 16 clinically diagnosed Saudi Arabian ADPKD patients, four *PKD1* mutations were identified in five patients and one *PKD2* mutation was identified in one patient. *PKD1* mutations included three protein truncating mutations (p.Gln3206*, p.Q3514*, and p.Arg2163*) and one missense mutation (p.Val4146Ile). All observed mutations were located within the duplicated region and were validated by Sanger sequencing. The *PKD2* mutation was a protein truncating mutation (p.Arg464*). *PKD1* and *PKD2* mutations were identified in six of nine (66.6%) patients with typical ADPKD presentations, while no *PKD1* or *PKD2* mutations were found in the seven patients with an atypical ADPKD feature (Table 1). No predicted pathogenic *PKD1 or PKD2* rare variants were observed in the 16 Saudi controls.

Six of the seven patients had no *PKD1* or *PKD2* mutation detected, but with a predicted pathogenic variant observed in genes involved in cystic pathways, including *PKHD1, PKD1L3, CFTR, EGF,* and *TSC2*, and four had atypical presentations with small kidney size and eGFR decline (P11, P12, P15, and P16). One patient carried a heterozygous *PKHD1* missense mutation (p.Arg1909Trp), one patient carried a *PKD1L3* nonsense mutation (p.Ser1168*), two patients carried a *CTFR* missense mutation (p.Ala120Thr), one patient carried a *EGF* missense mutation (p.Gly366Asp), and one patient carried a *TSC2* missense mutation (p.Ala985Val). Medical records of patients carrying these cyst-pathway genes did not display evidence of lung or liver disease. Progressive kidney disease was present in all the atypical patients, with five of the seven patients having CKD stage 5 and the remaining two having CKD stage 3 or lower. The patient with a rare predicted pathogenic *PKHD1* variant had been diagnosed with ADPKD based on CT imaging and a strong positive family history of the disease including the patient’s mother, three brothers, and daughter. The patient with a *PKD1L3* variant had a family history of the disease and had progressed to CKD stage 5 but the patient did not display evidence of liver disease. No patients were compound digenic heterozygotes, meaning no patient carried more than one rare variant in a cyst-pathway gene. Of the four ADPKD patients with no mutation detected in *PKD1, PKD2,* or any cyst-pathway gene, two were at CKD stage 5 but had small atrophic kidneys (P2 and P9), one was at stage 3 with typical ADPKD features (P4), and one had very mild disease with CKD stage 1 at age 74 (P8). Pedigree analysis revealed that 13 of the 16 ADPKD patients had a positive family history of ADPKD. It was not possible to confirm whether or not there was a positive family history in patients P8, P15, and P16 due to the unavailability of parental medical records as these patients had moved into the Eastern Province from rural areas in southern Saudi Arabia. While these three patients did not have a *PKD1* or *PKD2* mutation, P15 carried the *CFTR* mutation and P16 carried the *TSC2* mutation. No mutations were identified in ADPKD or cyst pathway genes in the control group.

Patients with *PKD1* and *PKD2* mutations demonstrated an earlier age of diagnosis (mean 31.7 ± 11.2 years vs. 43.0 ± 14.3 years), a higher total kidney volume, and higher MCIC, while patients with no *PKD1* or *PKD2* mutation detected had significantly older age of diagnosis, smaller total kidney volume, and lower risk MCIC.

Based on available clinical and genetic parameters (the presence of *PKD1* or *PKD2* mutation) the ‘predicting renal outcomes in ADPKD’ (PROPKD) score was calculated to predict the risk of ESRD and determine the median annual reduction of eGFR (Table 1). Of the six patients with either a *PKD1* or *PKD2* mutation, two patients were low risk, two were intermediate, and two were at high risk of a decline in kidney function.

## Discussion

ADPKD is the most prevalent inherited form of kidney disease and is observed in approximately 7–10% of patients with end-stage renal disease. Variable expressivity is common in ADPKD, with age at presentation and kidney disease severity greatly varying depending on the mutated gene, the mutation class, and the influence of other modifying genes [11]. Over 2,300 *PKD1* and 300 *PKD2* germline mutations have been identified in different populations, and the list is growing [12,30]. Clinical diagnosis of ADPKD is based upon the presence of kidney cysts on imaging in the context of a positive family history [31], typically with ultrasound, but also by CT or MRI. Positive family history is an important condition as it raises the pretest probability of ADPKD to 50%. Kidney enlargement is also an important consideration in ADPKD diagnosis, but is not a part of current diagnostic criteria. Moreover, diagnosis using imaging criteria alone is difficult in young patients (age < 30) as cysts and renal enlargement may not yet be apparent. Genetic diagnostics is thus especially valuable in evaluating young patients and those patients with no family history of ADPKD. Cases with equivocal or unusual presentations, early-onset, or severe presentations, and for improving risk stratification are emerging indications for genetic testing [31,32]. Furthermore, molecular diagnosis of ADPKD may contribute to family planning decisions.

Our study found *PKD1* and *PKD2* mutations in six of the nine (66.6%) patients with typical ADPKD features. No *PKD1* or *PKD2* mutation was detected in the seven patients with atypical features of ADPKD, calling the diagnosis into question. The Toronto Genetic Epidemiology Study of PKD (TGESP), a clinically ascertained cohort including primarily ADPKD patients of European descent, identified 60% (131 of 220 families) with *PKD1* mutations and 26% (57 of 220 families) with *PKD2* mutations using long-range PCR and Sanger sequencing [32]. Kinoshita and colleagues found putative pathological *PKD1* and *PKD2* mutations in 89% (90 of 101) of Japanese ADPKD patients with a long-range PCR and next-generation sequencing platform [33]. The lower rate of mutation detection in the current study is either due to technical artifacts leading to missed mutations in *PKD1* or *PKD2* or the presence of ADPKD phenocopies incorrectly diagnosed as ADPKD [34]. Somatic mosaicism is another potential cause for unidentified mutations, especially in the context of a patient without a family history [14]. While there have been reports of success using targeted gene panel sequencing in ADPKD, a recent report indicated low read depth and call quality over *PKD1* exons in the duplicated region using the Agilent TruSeq and SureSelect kits and Illumina HiSeq2000 platform [15–17]. We observed adequate read depth through both duplicated and non-duplicated regions of *PKD1* using the BGI pipeline, and all identified variants were validated by Sanger sequencing, but further validation to ensure the sensitivity of this approach is required. Exome sequencing technology is likely to continue to improve, but may be replaced with whole-genome sequencing strategies in the near future [35].

While previous reports have identified other variants of *PKD1* and *PKD2* in the Saudi population, we are reporting here for the first time five variants, namely c.12436G>A, c. 9616 C>T, c.6487C>T, c.10540C>T in *PKD1*, and c.1390C>T in *PKD2* in the Saudi population [36,37]. These variants have been previously identified in European populations [38,39]. Audrézet et al. [14] reported a number of variants in *PKD1* and *PKD2* in French and Tunisian populations. Mutations in *PKD1* and *PKD2* are associated with variable renal survival times in ADPKD patients [36,40]. The age of onset of ESRD in patients with *PKD1* mutations is approximately 58 years, whereas in patients with *PKD2* mutations, the age of onset is approximately 79 years. The age of onset of ESRD is also influenced by the type of *PKD1* mutation itself [36,40,41].

Protein-truncating *PKD1* mutations lead to a younger age at onset of ESRD than carriers of non-truncating mutations. In the present study, the four patients with a truncating *PKD1* mutation had a more severe kidney phenotype than the patient with a non-truncating mutation. The c.5725C>T mutation in the *PKHD1* gene, which is usually associated with ARPKD, has been reported in the Saudi population in association with ARPKD [42]. The subject carrying this mutation has a very strong family history of ADPKD (five members had ADPKD) and was at CKD stage 3. This patient did not present with any other organ disease. The c.3503C>G mutation in the *PKD1L3* gene, which is usually associated with sour ageusia, was identified in one subject. This subject carrying this mutation had one family member who had been diagnosed with ADPKD but the patient himself did not present with any other organ disease.

Numerous pathways have been implicated in cyst pathogenesis and progression. Epidermal growth factor (*EGF*) is a small mitogenic protein involved in normal cellular proliferation, acceleration of fluid secretion, renal electrolyte homeostasis, and expansion of renal cysts [43]. *EGF* protein is highly expressed in cyst epithelia of ADPKD patients, and primary cultures of cyst epithelia are hyperesponsive to mitogenic stimulation by *EGF*, which may indicate that *EGF* plays a role in promoting cyst formation [44]. One ADPKD patient in the present cohort was found to have a mutation in the *EGF* gene; however, the influence of this mutation on the phenotype of the disease could not be confirmed.

*CFTR* and purinergic channels in the apical membrane of the renal tubules mediate the conduction of chloride and bicarbonate across the plasma membrane, and it has been postulated that *CFTR* plays a role in cyst growth where inhibitors of *CFTR* retard cyst enlargement in animal model [45–47]. Moreover, it has also been postulated that inhibitors of *CFTR* may slow down the progression of polycystic kidney disease [45,46]. Two patients in the present cohort were found to have a mutation in the *CFTR* gene but the causative mutation of ADPKD in these two patients could not be identified.

Tuberous sclerosis complex (TSC) is an autosomal dominant disease characterized by facial angiofibromas, benign brain tumors, seizures, developmental delay, cardiac rhabdomyomas, pulmonary lymphangioleiomyomatosis, and renal angiomyolipomas [48]. TSC is caused by a pathogenic variant in either *TSC1* or *TSC2*. The *TSC2* gene is located adjacent to *PKD1* on chromosome 16p13.3. A contiguous deletion syndrome, where both *TSC2* and *PKD1* genes are deleted, has been reported to lead to a severe phenotype including signs and symptoms of TSC and PKD [49–51]. One patient in the present cohort was found to have a likely pathogenic variant in the *TSC2* gene, but no classical ADPKD mutation. This patient did not show any signs or symptoms of TSC in the brain, heart, lung, eye, or kidney.

One limitation of this study was that obtaining pedigree analysis of some patients was not possible as these patients had migrated to the Eastern Province from isolated rural areas in other provinces of Saudi Arabia where such information would be impossible to obtain. Furthermore, the patients themselves were unable to provide such information.

## Conclusions

Exome sequencing of DNA obtained from Saudi patients who had typical ADPKD revealed that mutations in *PKD1* and *PKD2* are the most common cause of typical ADPKD in this population. We also observed four rare variants that have the potential to contribute to an ADPKD phenotype. Further examination of exome sequencing techniques is required before clinical application of this technique in ADPKD diagnosis. However, genetic testing for ADPKD can improve diagnostic precision, informed prognosis, and support family planning.

## Supplementary Material

Supplemental Tables

Supplemental Figure
