# Supplementary material for: Exome sequencing of Saudi Arabian patients with ADPKD
Source: Ren Fail. 2019 Sep 5;41(1):842–9. doi: 10.1080/0886022X.2019.1655453 (PMC6735335; doi:10.1080/0886022X.2019.1655453)
Supplement: Supplemental Tables [file IRNF_A_1655453_SM2851.pdf]

Supplementary table 1: Exonic depth coverage of *PKD1*

Gene	Duplicated Region	Exon Number	Start	End	Read Coverage	Mean Depth
PKD1	Yes	1	2185476	2185690	80	1.71
	Yes	2	2169308	2169379	100	52.51
	Yes	3	2169115	2169186	100	136.42
	Yes	4	2168677	2168846	100	92.48
	Yes	5	2167792	2168463	100	32.59
	Yes	6	2167490	2167673	100	49.48
	Yes	7	2166834	2167054	100	24.53
	Yes	8	2166530	2166645	100	15.45
	Yes	9	2165993	2166119	100	32.07
	Yes	10	2165379	2165626	100	49.6
	Yes	11	2164171	2164926	100	61.65
	Yes	12	2163162	2163293	100	71.37
	Yes	13	2162789	2162964	100	165.27
	Yes	14	2162341	2162474	100	50.05
	Yes	15	2158253	2161872	100	65.95
	Yes	16	2157884	2158033	100	39.89
	Yes	17	2156806	2156949	100	129.85
	Yes	18	2156399	2156678	100	129.91
	Yes	19	2156092	2156305	100	32.86
	Yes	20	2155866	2156025	100	63.88
	Yes	21	2155323	2155475	100	80.22
	Yes	22	2154499	2154643	100	113.08
	Yes	23	2153267	2153896	100	93.52
	Yes	24	2152815	2152971	100	133.35
	Yes	25	2152382	2152634	100	83.19
	Yes	26	2152062	2152257	100	155.34
	Yes	27	2150397	2150567	100	187.29
	Yes	28	2150167	2150310	100	107.01
	Yes	29	2149862	2150072	100	133.91
	Yes	30	2149645	2149771	100	149.75
	Yes	31	2147869	2147985	100	60.76
	Yes	32	2147729	2147781	100	171.74
	Yes	33	2147320	2147504	100	125.94
	No	34	2147149	2147242	100	114.02
	No	35	2144093	2144211	100	84.28
	No	36	2143812	2144014	100	44.23
	No	37	2143545	2143739	100	150.53
	No	38	2142955	2143094	100	203.57
	No	39	2142481	2142593	100	112.08
	No	40	2142048	2142189	100	108.98
	No	41	2141782	2141907	100	52.02
	No	42	2141424	2141598	100	20.55
	No	43	2140885	2141175	100	24.27
	No	44	2140675	2140809	100	101.96
	No	45	2140286	2140591	100	91.96
	No	46	2139728	2140195	100	105.19

Supplementary table 2: Predicting renal outcomes in ADPKD in patients with PKD1 and PKD2 mutations based on PROPKD score

	P1	P3	P5	P6	P7	P10
**PROPKD Score ¥**	7	6	3	6	7	3
**Predicted risk of ESRD at age 60**	92%	61%	19%	61%	92%	19%
**PROPKD Risk category**	High	Intermediate	Low	Intermediate	High	Low
**Predicted Age at ESRD (years)**	49	57	70	57	49	70
**Median annual GFR decline (ml/min/m^2 per year)**	-4.4	-3.4	-2	-3.4	-4.4	-2
**Present Age (years)**	38	49	49	60	39	37
**CKD Stage**	4	5	5	5	3	1

(https://qxmd.com/calculate/calculator_437/propkd-score)
