# Supplementary material for: Exome sequencing of Saudi Arabian patients with ADPKD
Source: Ren Fail. 2019 Sep 5;41(1):842–9. doi: 10.1080/0886022X.2019.1655453 (PMC6735335; doi:10.1080/0886022X.2019.1655453)
Supplement: Supplemental Figure [file IRNF_A_1655453_SM2849.pdf]

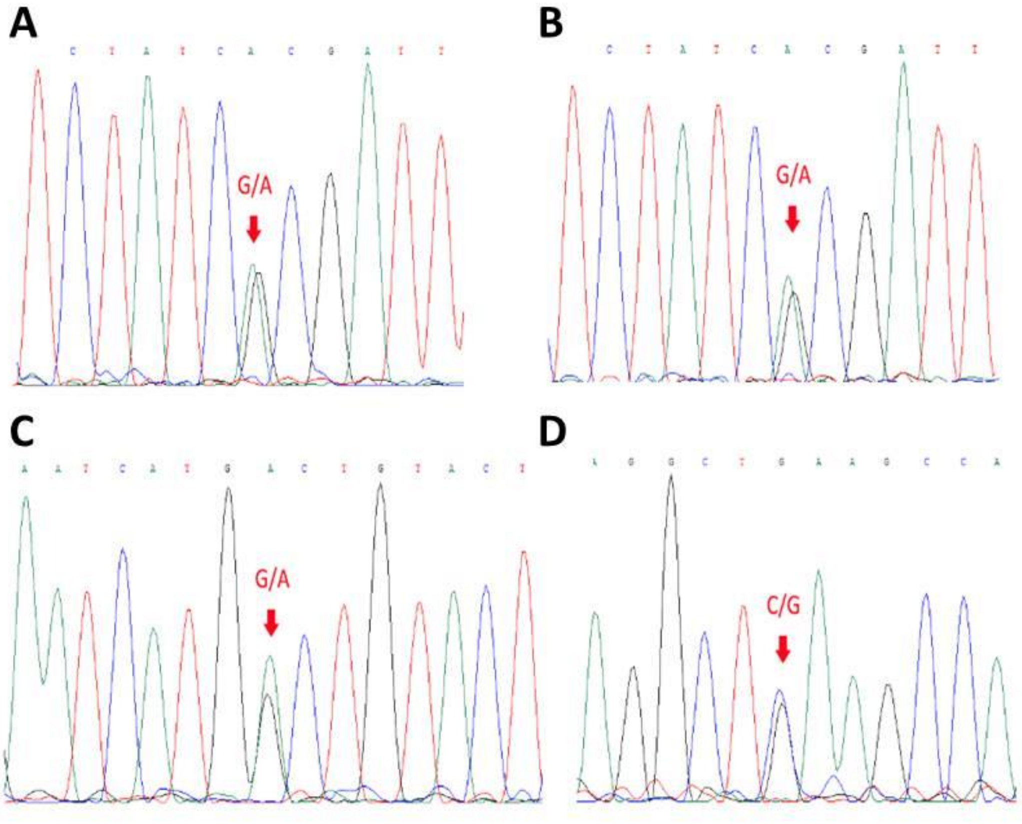

Supplementary Figure 1: Sanger sequencing validation of mutations identified by exome sequencing. Red arrows indicate the mutated alleles. (A,B) *CFTR* c.358G>A; (C) *EGF* c.1097G>A; (D) *PKD1L3* c.3503C>G
